# Utilizing a Hybrid Faculty-Guided, Self-Directed Study Model to Teach Immunology to First-Year Medical Students

**DOI:** 10.1007/s40670-025-02330-x

**Published:** 2025-02-26

**Authors:** Shirley Wong, Kencie Ely, Emily Weinschreider, Josh Levy, Edward Simanton, Dale Netski

**Affiliations:** 1 Kirk Kerkorian School of Medicine at University of Las Vegas, 1701 W. Charleston, Las Vegas, NV 89102 USA; 2 Department of Medical Education, Kirk Kerkorian School of Medicine at University of Nevada, Las Vegas, NV 89106 USA; 3 Office of Faculty Affairs, Kirk Kerkorian School of Medicine at University of Nevada, Las Vegas, NV 89102 USA

**Keywords:** Medical education, Self-directed learning, Hybrid Learning, Immunology, Asynchronous learning

## Abstract

This study evaluates the effectiveness of a hybrid, faculty-guided, self-directed learning model for teaching immunology to first-year medical students. It compares summative exam performance across five cohorts (2017–2021). The 2021 pilot course incorporated asynchronous videos, quizzes, and in-person sessions. Results show consistent performance across cohorts, except for the 2020 fully virtual cohort, which scored below the National Board of Medical Examiners (NBME) average. Student feedback highlighted the benefits of the hybrid model, particularly its flexibility and self-paced learning. These findings suggest that hybrid models can be used effectively to teach complex subjects like immunology with reduced in-person contact.

**Supplementary Information:**

The online version contains supplementary material available at 10.1007/s40670-025-02330-x.

## Background

The structure of medical school curricula poses distinct challenges, especially compared to the traditional undergraduate learning experience. During the pre-clinical years of medical school, course durations are condensed and have a much broader scope. Additionally, students with varied educational backgrounds enter medical school, making it challenging to develop courses that address all learning levels. One subject that exemplifies these challenges is immunology. This topic is not always included in pre-medical course requirements, so students enter medical school with diverse immunology knowledge. This varied knowledge base can pose a unique challenge when students encounter their first immunology course in medical school due to the vast amount of vocabulary and concepts, which can be difficult to grasp in the short time students have to learn the information.

Emerging trends in medical education emphasize learner autonomy, promoting a shift towards self-directed study and increased reliance on recorded lectures instead of live, in-person lectures [1]. Self-directed learning allows students to develop their own study plans based on their knowledge level and learning style. Furthermore, studies have shown that students prefer shorter online lectures and have rated third-party resources higher than traditional curricula in effectiveness, length, and quality [2]. This evolving educational landscape and shifting student attitudes may contribute to the growing number of students abstaining from attending in-person lectures during medical school [1].

The active-learning flipped classroom approach, which combines self-directed learning with instructor-guided instruction, has yielded positive outcomes in various health-related disciplines [3, 4]. One meta-analysis of 28 eligible studies demonstrated a significant increase in exam performance for medical students taught using a flipped classroom over a traditional approach [5]. This same study also showed that more respondents preferred flipped to traditional classrooms [5]. Potential explanations for these results included the ability to review pre-recorded videos as needed, more in-class active learning opportunities, and greater opportunities for instructors to provide feedback during in-class sessions [5].

Additionally, the COVID-19 pandemic forced medical schools to adopt a virtual classroom model, which taught institutions to use and develop more effective technology for online learning. This experience has allowed both institutions and students to be better equipped to handle mixed teaching formats. With the broader acceptance of virtual learning, we can be better prepared to seamlessly transition to an online classroom format if a similar situation were to arise, as well as cater to the evolving educational needs of students [6].

As part of a curricular evolution in our biomedical science program, we assessed the first- and second-year curricula for improvement opportunities at our new medical school. Four cohorts had completed at least their first year of medical school at the time of the assessment. In this short communication, we focus on the immunology course portion of our Introduction to Biomedical Sciences block, which occurs in the first semester of our curriculum.

The first three cohorts were taught immunology using a didactic, in-person format. This approach, however, posed challenges in engaging students with varying levels of immunology knowledge, leading to poor attendance. The fourth cohort, which matriculated during the COVID-19 pandemic in 2020, transitioned to a fully asynchronous, video-based emergency remote teaching plan approach due to the suspension of in-person classes [7]. In 2021, with the fifth cohort’s arrival and easing COVID-19 restrictions, we implemented a pilot 2-week intensive immunology course combining asynchronous video learning with an instructor-guided in-person component. This hybrid model incorporated lessons from the fully remote instruction of the fourth cohort and aimed to address the diverse educational backgrounds of the students.

To evaluate the pilot course’s effectiveness, we compared its structure and summative exam outcomes with those of the first four cohorts. We hypothesized that the hybrid approach would result in exam scores comparable to previous cohorts.

## Activity

This cohort study assesses the first 5 years (2017–2021) of our immunology curriculum by analyzing mean summative exam scores. The National Board of Medical Examiners (NBME) customized assessment services were used for summative exam questions. Cohorts experienced different instructional models: the 2017 – 2019 cohorts had 12 h of weekly scheduled class time, with attendance mandatory for the 2017 cohort (Charter Class) and optional for 2018–2019. In 2020, due to COVID-19, instruction was fully virtual, and the 2021 cohort had 4 h of mandatory in-person attendance.

Starting with the COVID cohort, student contact hours were significantly reduced with the introduction of the flipped classroom approach. Students watched pre-recorded basic immunology concept videos (3 h total across seven episodes) and read assigned textbook material. The COVID-19 cohort had asynchronous learning with a synchronous video call with the course director each Friday.

**Fig. 1:**
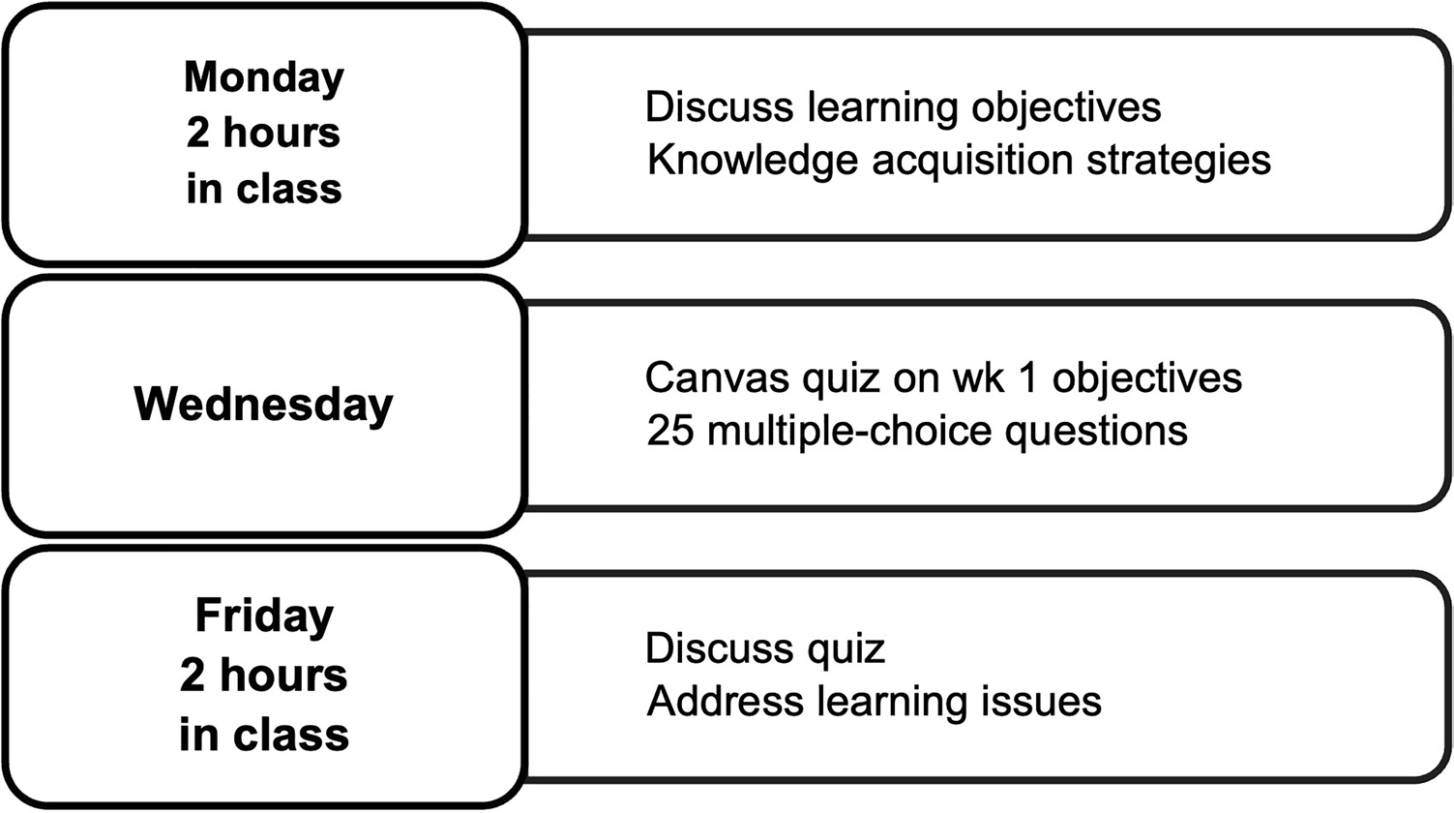
2021 pilot cohort weekly immunology schedule

The 2021 pilot course had a 2-week immunology schedule (Fig. 1). On Mondays, students attended a mandatory in-class session to discuss learning objectives, Wednesdays included an online multiple-choice quiz to assess concept mastery, and Fridays featured another mandatory in-person session to review quiz questions and address learning gaps.

## Results and Discussion

Mean summative immunology item scores for school exams and the NBME source exams were calculated for each cohort (Table 1). All cohorts, except the 2020 cohort, performed above the national NBME item score for those questions.

**Table 1:** Descriptives of mean immunology items correct for school exams versus the NBME source exams for the five medical student cohorts

Cohort year (*n*)	Immunology items on exam	% items correct	
		School exam (mean, SD)	NBME source (mean, SD)
2017 (60)	50	84.86, 15.78	81.50, 13.39
2018 (61)	50	90.06, 11.52	81.50, 13.39
2019 (61)	25	81.52, 15.78	78.48, 11.46
2020 (59)	20	81.75,11.65	88.10, 3.23
2021 (62)	25	83.56, 13.35	81.56, 9.76

Using one-way analysis of variance (ANOVA, SPSS version 28), exam performance means were compared between school cohorts, NBME source scores and the difference between each cohort, and the NBME source scores for that year. No significant differences were observed between school cohorts or NBME source scores. However, the difference between the school and NBME source scores was significant (data not shown). To identify which cohort(s) contributed to this significance, post hoc pairwise comparisons were conducted to analyze performance differences between the school and NBME source scores for each year. No significant differences were found between the 2021 pilot cohort and the other four cohorts (Table 2). However, the difference in performance between the school and NBME source scores for the 2020 COVID-19 cohort was significantly larger compared to the 2017 and 2018 cohorts (data not shown).

**Table 2:** Bonferroni post hoc pairwise comparisons of the 2021 pilot cohort versus other cohorts assessing differences in immunology exam performance of the student cohort relative to the NBME source scores for that year

Dependent variable	Cohort	Cohort	Mean difference (%)	Std. error (%)	*p*-value	95% CI
Difference in performance	2021	2017	− 1.36	2.96	1.000	(− 9.77, 7.05)
		2018	− 6.56	2.96	0.279	(− 14.97, 1.85)
		2019	− 1.04	3.42	1.000	(− 10.76, 8.68)
		2020	8.35	3.62	0.224	(− 1.96, 18.66)

Student feedback from the 2021 pilot course assessments was used to analyze the qualitative aspects of the curriculum. Of the 28 responses, 25 were positive, with most students praising the quality of the instructional videos. Additional feedback highlighted the value of quizzes in reinforcing learning.

Several conclusions can be drawn from the results of this study. Except for the 2020 COVID cohort, NBME performance remained consistent across the cohorts with various educational models. This finding supports our hypothesis that a faculty-guided, self-directed approach is effective for teaching immunology. This data further corroborates the results discussed in a scoping review by Tang et al., who found that medical students who underwent a flipped classroom approach, utilizing online videos before attending in-person lectures, performed equally or better on assessments than cohorts who attended live lectures only [8]. Of additional note, the lower performance of the 2020 cohort suggests that fully virtual curricula may be less effective than a hybrid or fully in-person model. In addition, the 2020 cohort began their medical education during COVID-19, and it is known that COVID cohorts generally performed below average on exams [9, 10].

This study also highlighted the observation that regardless of the amount of time spent in class or whether attendance was mandatory, there was no significant difference in exam performance between the 2017–2019 and 2021 cohorts. Each instructional model has its benefits and challenges, but this study showed that medical students can effectively learn complex topics with limited in-person time. However, for enhanced learning of immunology concepts, proper instructor guidance is necessary for students to succeed using pre-recorded instructional videos, textbooks, third-party resources, and minimal in-person sessions.

Student attitudes toward the pilot course were largely positive. Many appreciated the flexibility of pre-recorded lectures, which allowed them to learn at their own pace while covering the required material. Quizzes and in-class discussion sessions were also described as valuable for identifying knowledge gaps and facilitating deeper understanding. These attitudes complement current literature evaluating the advantages of mixed asynchronous and synchronous learning, including flexibility and convenience, self-pacing, time for reflection, and multimedia learning [11].

One consideration that is also important to acknowledge is the social aspect of in-person activities and lectures. As Dimassi et al. reported, students and faculty consider the time spent together in person that was lost due to the COVID-19 pandemic negatively impacted student growth and mental health [12]. In addition to acquiring knowledge, in-person class activities are crucial for interpersonal and professional development as well as student mental health. Although our study did not look specifically at the social aspect of our hybrid course model, we believe that an in-person component can help promote these skills as well as contribute to student wellness.

This study is limited by its small sample size, as it focused on a single institution and one cohort using the hybrid model. Additionally, we only examined one basic science course. While this study did not investigate the specific third-party resources used outside the classroom, another study conducted at our institution has examined this topic [13]. While the comparison between classes revealed no significant differences, other factors varied between the cohorts, including variations in block length and the number and pace of exams.

In conclusion, this study demonstrates that a faculty-guided, self-directed learning model, combining pre-recorded content and minimal in-person instruction, is effective for teaching immunology.

## Supplementary Information

Below is the link to the electronic supplementary material.

Supplementary file1 (DOCX 21 KB)
