# Supplementary material for: Utilizing a Hybrid Faculty-Guided, Self-Directed Study Model to Teach Immunology to First-Year Medical Students
Source: Med Sci Educ. 2025 Feb 26;35(3):1191–4. doi: 10.1007/s40670-025-02330-x (PMC12228903; doi:10.1007/s40670-025-02330-x)
Supplement: Supplementary file 1 — Supplementary file1 (DOCX 21 KB) [file 40670_2025_2330_MOESM1_ESM.docx]

**Title:** Utilizing a Hybrid Faculty-Guided, Self-Directed Study Model to Teach Immunology to First-Year Medical Students

**Journal:** Medical Science Educator

**Authors:** Shirley Wong^1^, Kencie Ely^1^, Emily Weinschreider^1^, Josh Levy^1^, Edward Simanton^2^, and Dale Netski^2,3^

**Affiliations:**

^1^ Kirk Kerkorian School of Medicine at University of Las Vegas, Las Vegas, Nevada, USA

^2^ Department of Medical Education, Kirk Kerkorian School of Medicine at University of Nevada, Las Vegas, Las Vegas, NV 89106, USA
^3^ Office of Faculty Affairs, Kirk Kerkorian School of Medicine at University of Nevada, Las Vegas, Las Vegas, NV 89102, USA

**Corresponding Author**: Dale Netski, Kirk Kerkorian School of Medicine at University of Las Vegas, 1701 W. Charleston, Las Vegas, NV 89106; Email: [dale.netski@unlv.edu](mailto:dale.netski@unlv.edu)

| **Supplemental Table 1** Scheduled course contact hours and other course contact hour requirements for the five cohorts from 2017-2021 during year 1 fall curriculum. | | | | |
| --- | --- | --- | --- | --- |
| Cohort Year (n) | Scheduled In-Class time Lecture (hrs/wk) | Problem Based Learning In-Class Time (hrs/wk) | Other concurrent courses (hrs/wk) | Total Time In-Class (hrs/wk) |
| 2017 (60) | 12 | 6 | 6 | 24 |
| 2018 (61) | 12 | 6 | 6 | 24 |
| 2019 (61) | 12 | 6 | 6 | 24 |
| 2020 (59) | 0 (Virtual) | 0 (Virtual) | 0 (virtual) | 0 (21hours virtual curriculum only) |
| 2021 (62) | 4 | 6 | 10 | 20 |

| **Supplemental Table 2** One-way ANOVA comparing exam performance means of the school cohorts, NBME Source scores and the difference between school performance and NBME source performance. | | | | | | |
| --- | --- | --- | --- | --- | --- | --- |
|  |  | Sum of Squares | Df | Mean Square | F | p-value |
| School Performance | Between Groups | 0.183 | 4 | 0.046 | 2.390 | 0.053 |
|  | Within Groups | 3.153 | 165 | 0.019 |  |  |
|  | Total | 3.336 | 169 |  |  |  |
| NBME Source Performance | Between Groups | 0.108 | 4 | 0.027 | 1.919 | 0.110 |
|  | Within Groups | 2.321 | 165 | 0.014 |  |  |
|  | Total | 2.429 | 169 |  |  |  |
| Difference (School – NBME) | Between Groups | 0.328 | 4 | 0.082 | 5.631 | <0.001 |
|  | Within Groups | 2.405 | 165 | 0.015 |  |  |
|  | Total | 2.733 | 169 |  |  |  |
